# Effects of high-altitude hypoxic environment on colonic inflammation, intestinal barrier and gut microbiota in three-way crossbred commercial pigs

**DOI:** 10.3389/fmicb.2022.968521

**Published:** 2022-09-08

**Authors:** Chengzeng Luo, Guangming Sun, Jiujun Duan, Haiyu Han, Ruqing Zhong, Liang Chen, Basang Wangdui, Yanbin Zhu, Zirong Wang, Hongfu Zhang

**Affiliations:** ^1^College of Animal Science, Xinjiang Agricultural University, Urumqi, China; ^2^State Key Laboratory of Animal Nutrition, Institute of Animal Science, Chinese Academy of Agricultural Sciences, Beijing, China; ^3^Institute of Animal Husbandry and Veterinary Medicine, Tibet Academy of Agriculture and Animal Husbandry Science, Lhasa, China; ^4^Tibet Changdu Animal Husbandry General Station, Changdu, China

**Keywords:** three-way crossbred commercial pigs, high-altitude hypoxic environment, intestinal barrier, gut microbiota, inflammatory cytokines, short-chain fatty acids

## Abstract

In recent years, the three-way crossbred commercial pigs are extensively cultured in Tibet. However, there have been few studies about the effect of high-altitude hypoxic environment on intestinal health of them. Therefore, we selected Tibetan pigs (TP) and the three-way crossbred commercial pigs (CP-H) living in the Tibet (3,500–3,700 m in altitude) as a positive control group and treatment group, respectively. The three-way crossbred commercial pigs (CP-L) living at altitudes 800–1,000 m sea level were selected as a negative control group. The colonic chyme, colonic mucosa, colonic tissue and serum samples were collected for the detection of gut microbiota and intestinal inflammation. The results showed that high-altitude hypoxic environment promoted the occurrence of colonic inflammation, disrupted the colonic barrier to some extent. And Hematoxylin–Eosin (HE) staining revealed that mild inflammatory cell infiltration was observed in colon of CP-H. 16S rRNA gene sequencing revealed that the microbial community composition of CP-H was changed compared with CP-L. Gut bacterial communities formed distinctly different clusters in principal coordinates analysis (PCoA) space, and Chao 1 index of CP-H was also decreased. At the genus level, *Terrisporobacter* showed greater enrichment in the CP-H than lower-altitude pigs. *Colstridium-sensu-stricto-1* showed lower enrichment in the CP-H than lower-altitude pigs. However, the concentration of valeric acid in colonic chyme of CP-H was higher than CP-L and TP. Correlation analysis indicated that *Terrisporobacter* was positively associated with the relative mRNA expression level of *IL-1β* and the content of lipopolysaccharide (LPS), and was negatively correlated with the relative mRNA expression level of *IL-10*. The *Streptococcus* was positively associated with the concentrations of valerate. In summary, high-altitude hypoxic environment changed compositions of gut microbiota, promoted the occurrence of colonic inflammation, and disrupted intestinal barrier of the three-way crossbred commercial pigs.

## Introduction

In recent years, with the rapid development of society and the increasing demand for a better life, the demand of people living in Tibet is more diversified for meat products, especially for pork of three-way crossbred commercial pigs. To resolve this contradiction, the breeding of three-way crossbred commercial pigs was rapidly implemented under the unified leadership of the local government. According to statistics of the local government, in recent years, the scale of breeding for three-way crossbred commercial pigs has been more than 300,000 in Tibet, and the scale of breeding is steadily increasing.

At present, high-altitude hypoxic environment has been shown to increase the development of inflammation throughout the body or multiple-organ dysfunction possibly (Pham et al., 2021). For example, high-altitude hypoxic environment induces acute mountain sickness, high altitude pulmonary edema, and high altitude cerebral edema (Purushothaman et al., 2011), and can even also affect the growth and intestinal health (Jensen and Moore, 1997; Xu et al., 2014). In addition, it has also been shown that inflammatory signaling in response to high altitude hypoxic environment exist an adaptive mechanism (Pham et al., 2021). Tibetan pigs, also known as ginseng pigs, are the indigenous breed native to the Qinghai-Tibet Plateau. In order to adapt to high-altitude environments, Tibetan pigs have developed unique physiological mechanisms during the long process of evolution and have acquired stable structural and functional characteristics (Yang et al., 2021). In the last few years, the commercial pigs had also been raised in Qinghai-Tibet Plateau because of its higher growth rate, higher feed efficiency, and superior meat yield (Zhang et al., 2018). However, the three-way crossbred commercial pigs were purchased from plain areas of China soon after weaning. They may face immense living challenges from high altitude hypoxic environment due to environmental changes (Lanikova et al., 2017).

As is well known, the gut is an important physiological function organ (Brestoff and Artis, 2013). The microbiota located in the gut exerts important functions for gut homeostasis and host health. Studies have proven that intestinal microbiota is strongly linked to intestinal barrier function and systemic inflammation (Pickard et al., 2017). For example, enteric pathogen *C. rodentium* is a major hallmark of diarrheal disease for mice, its expansion can result in dramatic colonic mucosal hyperplasia and a local Th1 inflammatory response. *Clostridium difficile* infection can result in the occurrence of inflammatory bowel disease (Sokol et al., 2018). In contrast, the abundances of intestinal beneficial bacteria are positively related to the intestinal health, such as *Lactobacillus* and *Bifidobacterium* maybe improve intestinal barrier function and reduce intestinal inflammation (Wu et al., 2020). Meanwhile, gut microbiome is also influenced by many factors, such as genetic, foods and environmental factors (Goodrich et al., 2017), and it has been the widely validated. In addition to above factors, high-altitude environments affect the composition and function of the gut microbiota (Suzuki et al., 2019). At high altitude, there is a decrease in the barometric pressure and a consequent reduction in the oxygen partial pressure, which will lead to the increased abundances of the obligate anaerobes (Moreno-Indias et al., 2015). At present, a few studies have explored the microbial community composition of mammals living at different altitudes, including ruminants (Zhang et al., 2016), humans (Lan et al., 2017), and pikas (Li et al., 2016). However, the studies about that the effects of high-altitude hypoxic environment on colonic inflammation, intestinal barrier and gut microbiota in three-way crossbred commercial pigs have so far hardly been explored. Therefore, we selected Tibetan pigs and three-way crossbred commercial pigs living at 3500–3700 m as a positive control group and treatment group in the present trial, respectively. Moreover, three-way crossbred commercial pigs living at 800–1000 m were also selected as negative control group. We used 16S rRNA gene sequencing to analyze the microbiota between Tibetan pigs and three-way crossbred commercial pigs, analyzing the relationship between gut microbiota and intestinal parameters such as intestinal inflammation and intestinal barrier. This study thus sought to explore the effects of high-altitude hypoxic environment on gut microbiota and gut health of pigs.

## Materials and methods

### Ethics statement

All procedures in the current study including animal experiments and sample collection were approved by the Experimental Animal Welfare and Ethical Committee of the Institute of Animal Science, Chinese Academy of Agricultural Sciences (No. IAS2021-241).

### Animal and sample collection

Tibetan pigs (TP) and three-way crossbred commercial pigs (CP-H) were cultured under the research farm (Shannan District, Tibet, China) of Institute of Animal Science and Veterinary, Tibet Academy of Agricultural and Animal Husbandry Sciences (3,500–3,700 m above sea level). Three-way crossbred commercial pigs (CP-L; with the same genetic background as three-way crossbred commercial pigs above) were cultured under a commercial pig farm in Shanxi Province (800–1,000 m above sea level). All the pigs were reared according to the feeding standards and water *ad libitum* (Huang et al., 2021), and were fed with the same standard diet (Table 1). The pigs were euthanized for 6 randomly selected pigs per group when reached slaughter weights in this study. The blood was collected in order to obtain serum. Fresh colonic chyme and mucosa were fleetly collected, immersed in liquid nitrogen immediately, then stored at-80°C until further analysis. Finally, the colonic tissue samples were taken out and fixed in 4% formaldehyde.

**Table 1:** Composition and nutrient levels of diet.

Ingredient composition, %	Diet
Corn	66.40
Soybean meal Full-fat soybean	15.50
Wheat bran Soybean meal	15.00
Limestone Dried whey	1.00
CaHPO_4_ Soybean oil	0.50
NaCl CaHPO4	0.30
L-Lys (70%) NaCl	0.20
Choline chloride	0.10
Premix^1^ Choline chloride Limestone	1.00
Total Choline chloride	100.00
Calculated nutrient levels，% Lysine HCl	
CP Met	14.83
DE, MJ/kg Thr	13.90
NE, MJ/kg Glucose	10.37
SID Thr	0.43
SID Trp Premixb	0.13
SID Lys	0.75
SID Met DE (MJ/kg)	0.23
Ca CP	0.56
TP Lys	0.46
STTD P Met	0.25

1 Provided the following quantities per kg of diet: vitamin A, 9,140 IU; vitamin D3, 4,405 IU; vitamin E, 11 IU; menadione sodium bisulfite, 7.30 mg; riboflavin, 9.15 mg; D-pantothenic acid, 18.33 mg; niacin, 73.50 mg; choline chloride, 1,285 mg; vitamin B_12_, 200 ug; biotin, 900 ug; thiamine mononitrate, 3.67 mg; folic acid, 1,650 ug; pyridoxine hydrochloride, 5.50 mg; I, 1.85 mg; Mn, 110.10 mg; Cu, 7.40 mg; Fe, 73.50 mg; Zn, 73.50 mg; Se, 500 ug.

### Tissue sample and intestinal morphology

The colon tissues were immersed and fixed with 4% formaldehyde. Then, they were removed from formalin and embedded in paraffin. Subsequently, the paraffin blocks were sectioned at 5 μm thick sections using a semi-automatic microtome (LONGSHOU, China). Next, they were stained with hematoxylin and eosin according to methods of Luo et al. (2021), and observed under optical microscope and calculated histologic colitis score according to a pathology score system. Methods of pathology score system are described briefly below. Inflammation score was graded from 0 to 3 depending on the severity of the inflammation and infiltration of immune cells. Inflammation extent was graded from 0 to 3 with regard to the width of colon membrane affected by colitis including mucosa, sub-mucosa and transmural layers. Crypt damage was graded from 0 to 4 considering the damage of crypt and epithelial cells. Histologic colitis score was calculated by summing inflammation severity, inflammation extent and crypt damage.

### Concentrations of SCFAs

Approximately 1 g of the colonic chyme was collected and immersed in 10 ml of ddH_2_O in 15-mL screw capped vials, after shaked for 30 min, refrigerated at 4°C overnight. The mixture was centrifuged at 10,000 rpm for 10 min for the concentration analysis of SCFAs. Concentrations of SCFAs were determined by gas chromatography according to the method of Wu et al. (2016).

### DNA extraction, 16S rRNA gene amplification, sequencing and analysis

Approximately 0.5–1 g the colonic chyme was collected, respectively, from each sample, and microbial community genomic DNA was extracted microbial community genomic DNA according to the manufacturer’s instructions of the E.Z.N.A.® soil DNA Kit (D5625-02, Omega Bio-Tek Inc., Norcross, GA, United States). Then it was stored at-80°C until the time of analysis. In addition, the purity and DNA concentration were checked by 1% agarose gel electrophoresis and NanoDrop2000 spectrophotometer (Thermo Fisher Scientific, Waltham, MA, United States) separately. The V3-V4 regions of bacterial 16S rRNA gene were amplified with the following primer set: 338F (5′-ACTCCTACGGGAGGCAGCAG-3′) and 806R (5′-GGACTACHVGGGTWTCTAAT-3′). The reaction system, determination of amplified fragments and purification were performed according to methods of Luo et al. (2021). The raw microbial sequence data were analyzed and processed by the Majorbio Bio-Pharm Technology Co. Ltd. (Shanghai, China). The sequences were analyzed and assigned to operational taxonomic units (OTUs; 97% identity). What is more, the alpha-diversity, whose coverage was based on the Chao 1 and Shannon index within each sample was generated by QIIME (Version 174 1.7.0; Qingsen et al., 2017), and beta diversity was estimated by computing the unweighted Unifrac distance and visualized using PCoA.

### Genes expression

Total RNA of colonic mucosa was extracted using TransZol reagent (Ambion, UT, United States). Then the integrity and quality of total RNA were tested by electrophoresing on a 1.0% agarose gel and the Nano Drop® ND-1000 spectrophotometer (Nano-Drop Technologies, Wilmington, DE, United States), respectively. The cDNA was synthesized by a reverse transcription kit. The cDNA was stored at-20°C for quantitative real-time PCR (qRT-PCR). All primers were designed according to the method of Luo et al. (2019), and were synthesized by Sangon Biotech Corporation (Sangon Biotech, China). The primer sequences were listed in Table 2. qRT-PCR assays were performed according to the method of Luo et al. (2021). The expression levels of all were calculated by 2^−ΔΔCT^ methods (Luo et al., 2019).

**Table 2:** The nucleotide sequences of primer.

Target gene	Forward sequence (5′- 3′)	Reverse sequence (5′- 3′)
ZO-1	CTCCAGGCCCTTACCTTTCG	GGGGTAGGGGTCCTTCCTAT
Occludin	CAGGTGCACCCTCCAGATTG	TATGTCGTTGCTGGGTGCAT
TNF-α	TAAGGGCTGCCTTGGTTCAG	AGAGGTTCAGCGATGTAGCG
IL-1β	ATTCAGGGACCCTACCCTCTC	CTTCTCCACTGCCACGATGA
P65	CGAGAGGAGCACGGATACCA	CCCGTGTAGCCATTGATCTTG
IL-6	TGAGGCAAAAGGGAAAGA	GCGCAGGATGAGAATGA
IL-10	TCGGCCCAGTGAAGAGTTTC	GGAGTTCACGTGCTCCTTGA
GAPDH	GGGCATGAACCATGAGAAGT	GGGCATGAACCATGAGAAGT
β-actin	GCGTAGCATTTGCTGCATGA	GCGTGTGTGTAACTAGGGGT

### LPS, DAO and D-lactide

LPS in serum was measured using the commercially available tachypleus amebocyte lysate kit (Chinese Horseshoe Crab Reagent Manufactory Co., Ltd., Xiamen, China) according to the method of quantitative Chromogenic Limulus Amebocyte Lysate assay. DAO and D-lactide in serum were measured by kit from Nanjing Jiancheng Bioengineering Institute (Nanjing, China).

### Statistical analysis

Morphology of colon, colonic permeability, mRNA expression, and concentrations of SCFAs date were analyzed using SPSS (23.0). The correlation matrix between the bacterial species, and intestinal barrier, inflammatory cytokines, SCFAs and blood biochemical parameters were generated using Pearson’s correlation coefficient. The *p*-value below 0.05 was considered significant, and absolute value of r higher than 0.5 was considered a strong correlation factor (Cianga et al., 2021). GraphPad 8.0 was used to draw all the above data. Finally, the results were presented as means ± SEM, and with ^*^ indicating a statistically significant difference (*p <* 0.05), ^**^ indicating a highly significant difference (*p <* 0.01).

## Results

### Histologic colitis score from colonic sections and LPS, DAO, and D-lactide in serum

**Figure 1:**
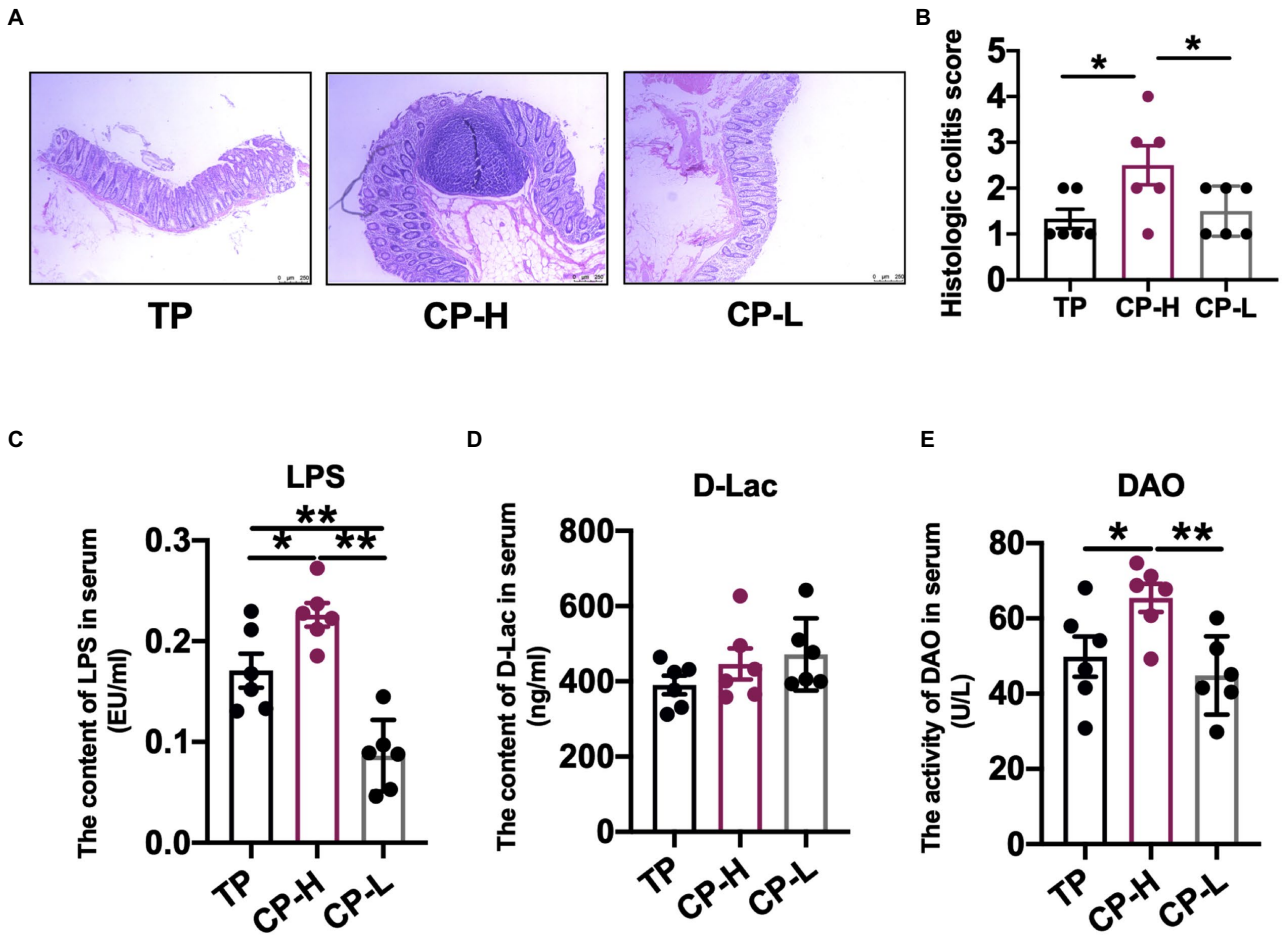
Effects of high-altitude hypoxic environment on intestinal tissue and LPS, DAO and D-lactide in serum of pig. TP: positive control group, CP-H: treatment group, CP-L: negative control group. **(A)** HE-stained pathological sections of colonic tissue from pigs of groups. **(B)** Histologic colitis score of pigs. **(C)** The content of LPS in serum of pigs. **(D)** The content of D-Lac in serum of pig. **(E)** The activity of DAO in serum of pigs. Data are expressed as mean ± SEM (*n* = 6). ^*^ indicating a statistically significant difference (*p <* 0.05), ^**^ indicating a highly significant difference (*p <* 0.01).

The morphological observations and pathological slices of colon were shown in Figure 1A. Mild inflammatory cell infiltration was observed in colon of CP-H. There was no inflammatory cell invasion in colon of TP and CP-L. The histologic colitis score was shown in Figure 1B. The histologic colitis score of CP-H was significantly increased compared with the TP and CP-L (*p <* 0.05).

There were no significant differences in the level of D-Lac in serum (Figure 1D). The activity of DAO and the level of LPS in serum of CP-H were significantly higher compared with TP and CP-L (*p <* 0.05; Figures 1C,E).

### Concentrations of SCFAs in colonic chyme

**Figure 2:**
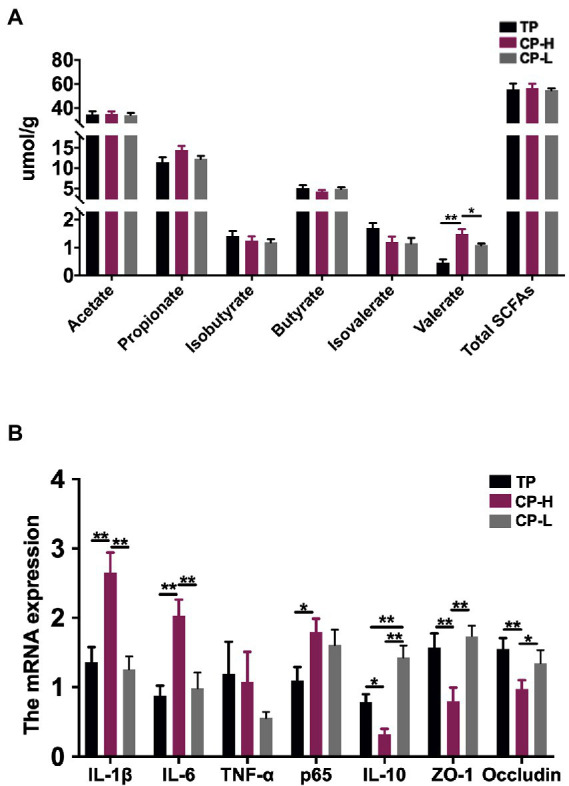
Effects of high-altitude hypoxic environment on intestinal inflammation and concentrations of SCFAs in the colonic chyme of pigs. TP: positive control group, CP-H: treatment group, CP-L: negative control group. **(A)** The concentrations of Acetate acid, Propionic acid, Isobutyric acid, Butyric acid, Isovaleric acid, Valeric acid and total SCFAs in the colonic chyme of pigs. **(B)** The mRNA expression levels of *IL-1β*, *IL-6*, *TNF-α*, *p65*, *IL-1*0, *ZO-1* and *Occludin* in colonic mucosa of pigs. Data are expressed as mean ± SEM (*n* = 6). ^*^ indicating a statistically significant difference (*p <* 0.05), ^**^ indicating a highly significant difference (*p <* 0.01).

The concentrations of acetic acid, propionic acid, isobutyric acid, butyric acid, and isovaleric acid in colonic chyme were not statistically significant (Figure 2A). In addition, concentrations of total SCFAs also revealed no significant differences (Figure 2A). However, the concentration of valeric acid in colonic chyme of CP-H was higher than CP-L and TP (*p* < 0.05; Figure 2A).

### Intestinal inflammation in colonic mucosa

Relative mRNA expression levels of *IL-1β* and *IL-6* in colonic mucosa of CP-H were higher relative to TP and CP-L (*p <* 0.01), and no significant differences were found between the two control groups (Figure 2B). Relative mRNA expression level of *p65* in CP-H was higher relative to TP (*p <* 0.05), but there were no significant differences between CP-H and CP-L (Figure 2B). Relative mRNA expression levels of *IL-10, ZO-1*, and *Occludin* in colonic mucosa of CP-H were lower relative to TP and CP-L (*p <* 0.05; Figure 2B). Furthermore, we also found that relative mRNA expression level of *IL-10* of CP-L was significantly higher than TP.

### Composition of gut microbes in colonic chyme

**Figure 3:**
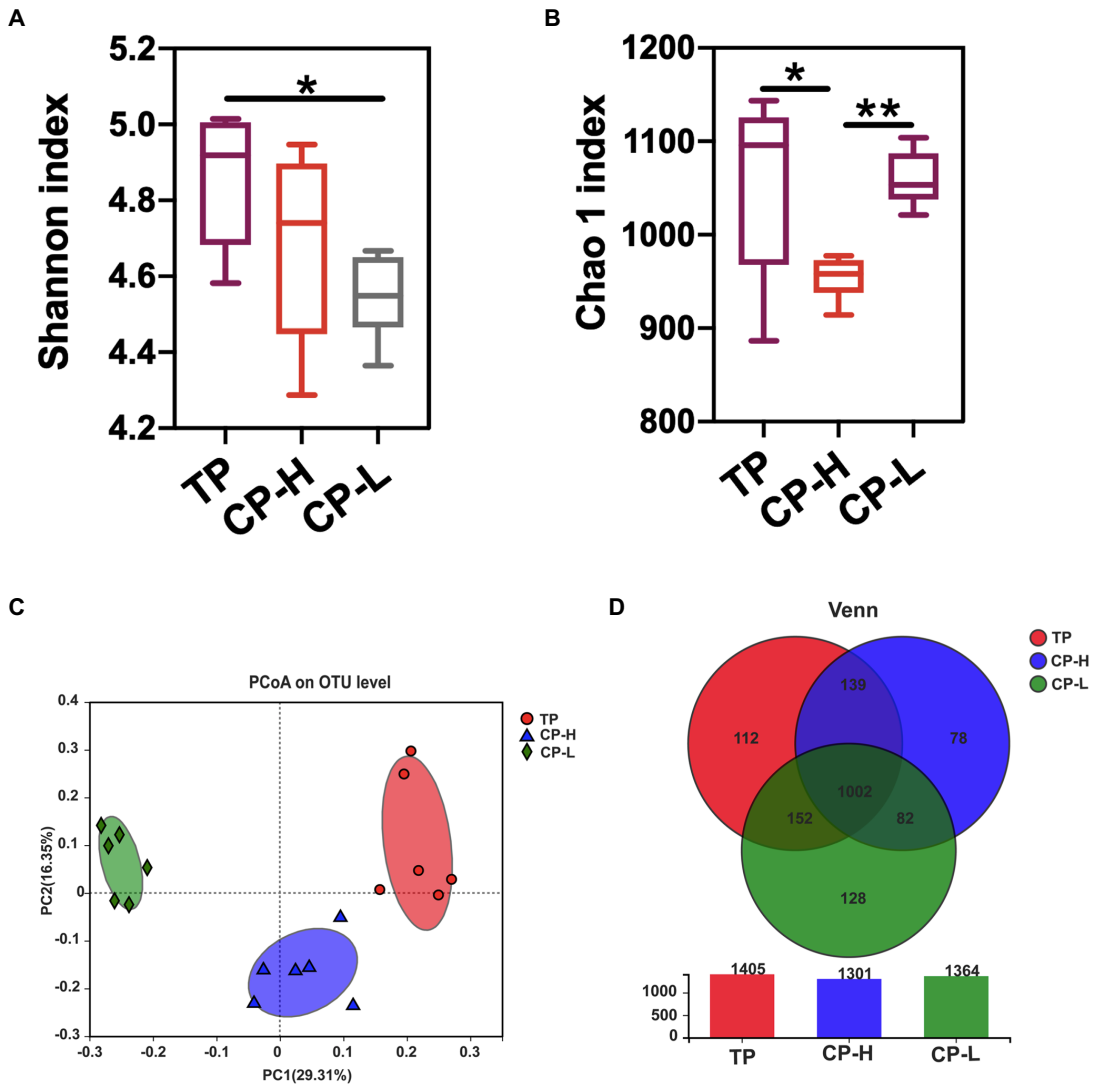
Effects of high-altitude hypoxic environment on gut microbiota diversity of pigs. TP: positive control group, CP-H: treatment group, CP-L: negative control group. **(A)** The Shannon index of microbiota in colonic chyme. **(B)** The Chao 1 index of microbiota in colonic chyme. **(C)** PCoA analysis of microbiota in colonic chyme at the OUT level. **(D)** The Wayne figures of microbiota in colonic chyme. Data are expressed as mean ± SEM (*n* = 6). ^*^ indicating a statistically significant difference (*p <* 0.05), ^**^ indicating a highly significant difference (*p <* 0.01).

To further assess whether differences in gut microbiota are the causal factor for the differences in gut barrier among TP, CP-H, and CP-L. The fresh colonic chyme was obtained from TP, CP-H, and CP-L, and 16 s rRNA gene sequencing analysis was performed. Shannon index of colonic chyme in TP was higher than those in CP-H and CP-L, and a statistically significant difference was found between TP and CP-L (*p <* 0.05; Figure 3A). Chao 1 index of CP-H was significantly lower than other groups (*p <* 0.05; Figure 3B). The PCoA indicated that TP, CP-H, and CP-L were distinctly clustered separately in distribution of microbiota at the colonic chyme (Figure 3C). There were 1,405, 1,301, and 1,364 operational taxonomic units (OTUs) obtained from TP, CP-H, and CP-L, respectively, of which 1,002 were common OTUs among the three experimental groups (Figure 3D).

**Figure 4:**
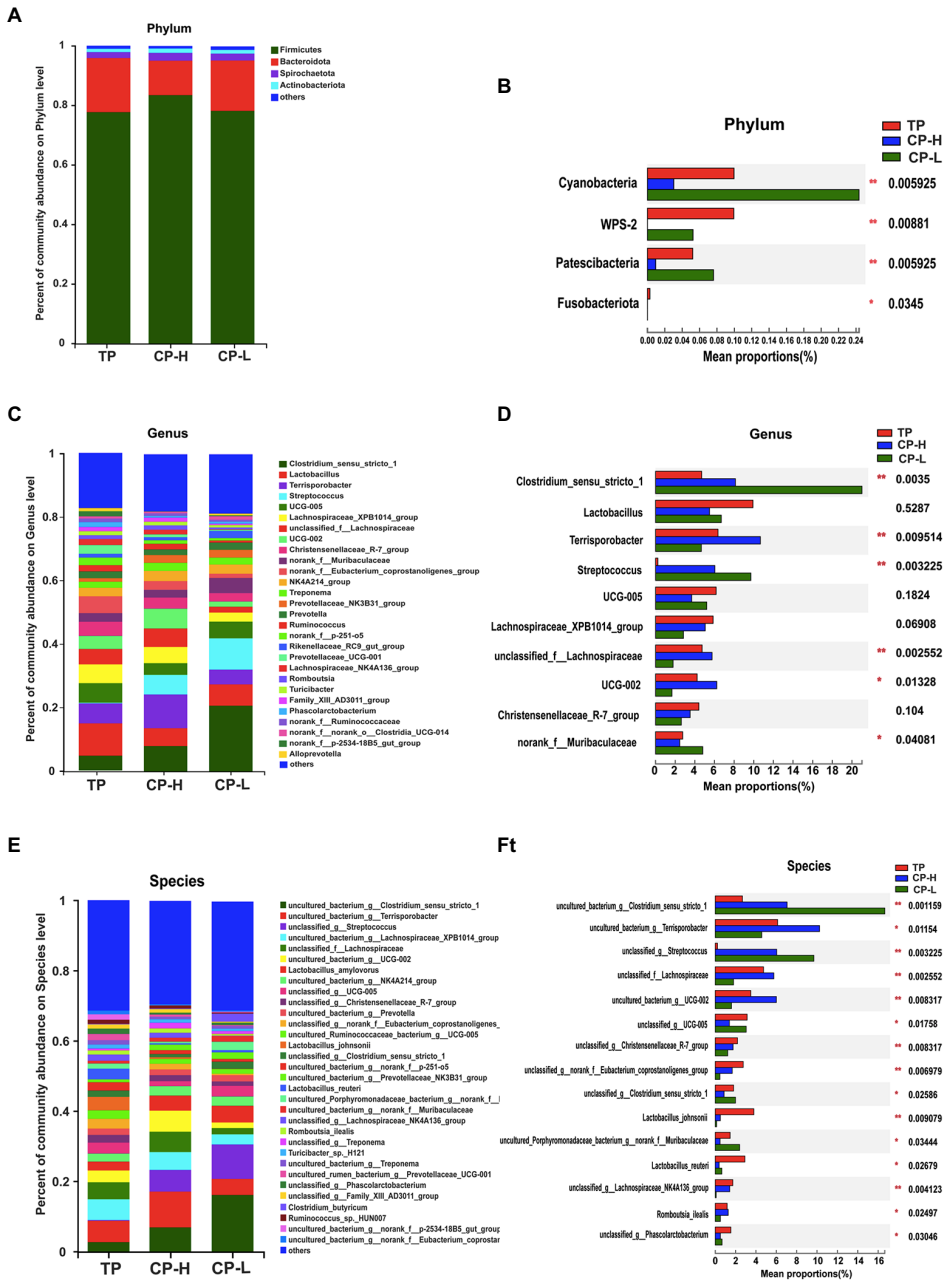
Effects of high-altitude hypoxic environment on gut microbiota community composition in the colonic chyme of pigs. TP: positive control group, CP-H: treatment group, CP-L: negative control group. **(A)** The relative abundances of microbiota in colonic chyme at the phylum level. **(B)** Analysis of variance the gut microbiota with significant differences at the phylum level. **(C)** The relative abundances of microbiota in colonic chyme at the genus level. **(D)** Analysis of variance about relative abundance of the top 10 genera in the three groups. **(E)** The relative abundances of microbiota in colonic chyme at the species level. **(F)** Analysis of variance about relative abundance of the top 15 genera with significant differences at the species level. Data are expressed as mean ± SEM (*n* = 6). ^*^ indicating a statistically significant difference (*p <* 0.05), ^**^ indicating a highly significant difference (*p <* 0.01).

Microbial community composition at the phylum, genus, and species level of the three groups were presented in Figures 4A–F. The results showed that colonic chyme samples comprised four major phyla including *Firmicutes*, *Bacteroidota*, *Spirochaetota*, and *Actinobacteria* (Figure 4A). *Firmicutes* and *Bacteroidetes* were the most predominant phyla in the colons of TP, CP-H, and CP-L (Figure 4A). In addition, there were also significant differences in abundance of *Cyanobacteria*, *WPS-2, Patescibacteria,* and *Fusobacteriota* among three breeds (*p <* 0.05; Figure 4B). At the genus level, the top three most abundant genus in three groups, in turn, were *Colstridium-sensu-stricto-1*, *Lactobacillus*, and *Terrisporobacter* (Figure 4C). Composition of the gut microbiota was further analyzed at the species level (Figures 4E,F). The *Lactobacillus_johnsonii* and *Lactobacillus_reuteri* were significantly enriched in TP (Figures 4F).

**Figure 5:**
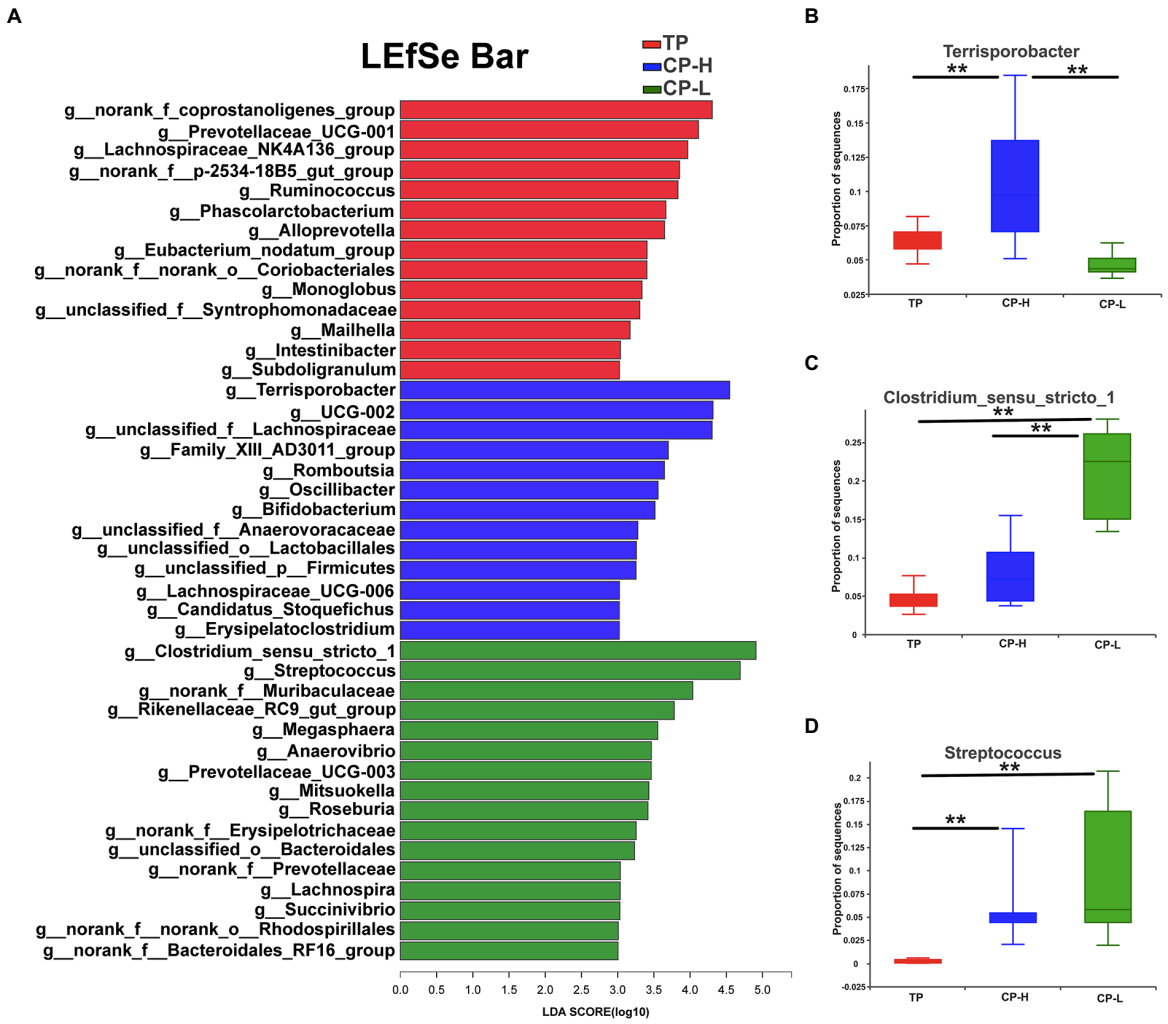
The differentially abundant taxa among the three groups by LEfSe analysis, and analysis of variance about some specific bacteria genera. TP: positive control group, CP-H: treatment group, CP-L: negative control group. **(A)** LEfSe analysis of microbiota in colonic chyme. **(B)** Analysis of variance about *Terrisporobacter*. **(C)** Analysis of variance about *Clostridium_sensu_stricto_1*. **(D)** Analysis of variance about *Streptococcusare*. Data are expressed as mean ± SEM (*n* = 6). ^**^ indicating a highly significant difference (*p <* 0.01).

LEfSe analysis was performed and presented as LDA score ≥ 3.0 to further explore the differences in microbiota composition between the three groups. The results were showed in Figure 5A. A total of 43 biomarkers in TP, CP-H, and CP-L. There were 14, 13, and 16 biomarkers in TP, CP-H, and CP-L, respectively. Notably, *Terrisporobacter* was enriched in colonic chyme of CP-H, *Colstridium-sensu-stricto-1* and *Streptococcus* were enriched in colonic chyme of CP-L. The relative abundance of the three genera varied among the three groups (*p <* 0.05; Figures 5B–D).

### Correlation between microbial communities and gut barrier

**Figure 6:**
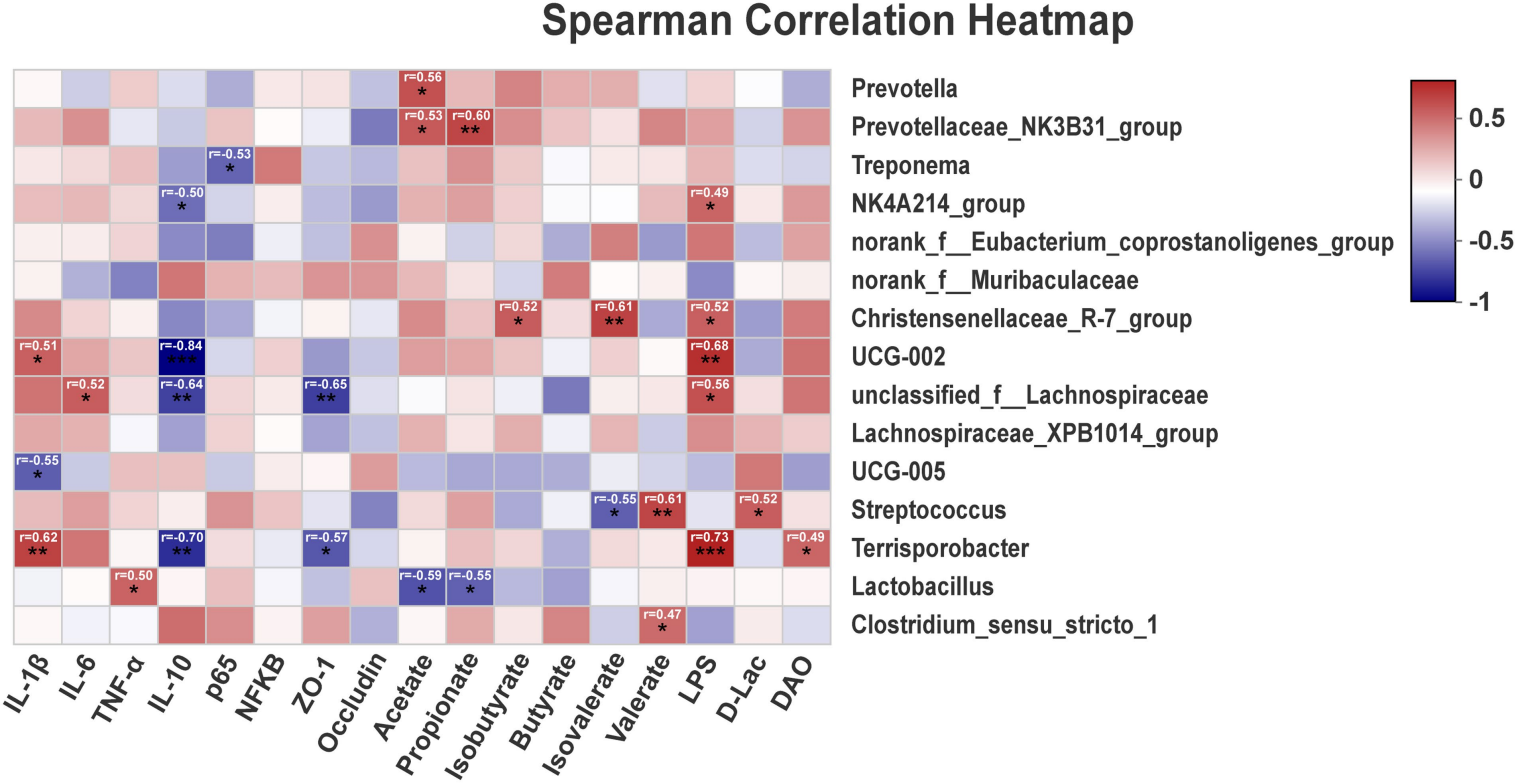
The correlation of intestinal microbiota, intestinal barrier, SCFAs, inflammatory cytokines, and blood biochemical parameters. r = Pearson correlation coefficient; *p* = significance level, ^*^p < 0.05, ^**^p < 0.01, ^***^p < 0.001. The *p*-value below 0.05 was considered significant, and absolute value of r higher than 0.5 was considered a strong correlation factor.

Correlations between metabolites and the top 15 genus between TP, CP-H, and CP-L were obtained *via* Pearson’s correlation analysis. As shown in Figure 6, the results showed that the relative abundance of *Terrisporobacter* was positively associated with the relative mRNA expression level of *IL-1β*, the content of LPS, and was negatively correlated with the relative mRNA expression level of *IL-10* (Figure 6). The relative abundance of *Streptococcus* was positively associated with the concentration of valerate (Figure 6).

## Discussion

Intestinal barrier, including ecological barrier, mechanical barrier, and immunity barrier, plays an integral role in maintaining gut homeostasis. Among them, the intestinal mechanical barrier is essentially a defensive layer composed of intestinal mucosal epithelial cells and tight junctions between cells and the bacterial membrane, and can effectively prevent intestinal injury (Odenwald and Turner, 2017). Break of mechanical barrier often show obvious mucosal ulceration, loss of crypt, goblet cells and epithelial damage, neutrophil infiltration and causes inflammation (Turner, 2009; Li et al., 2019a). In this study, the occurrence of local mild inflammatory cell infiltration was observed in the colon of CP-H group. The level of D-Lac, LPS and the activity of DAO in serum of CP-H group were higher compared with CP-L group. Moreover, we also found that relative mRNA expression levels of *IL-1β, IL-6* and *p65* in colonic mucosa of CP-H group were higher relative to CP-L group. These data evidenced that the colon of CP living in plateau were in the inflamed state, and the intestinal barrier was disrupted to the certain extent. This situation may be related to the altitude at which the CP were located (Yi et al., 2021). It has been reported that upon exposure to high altitude, oxygen deficit and oxidative stress can occur (Földes-Papp et al., 2005; Pichler Hefti et al., 2016). Subsequently, inflammatory factors are released in response to hypoxia and oxidative stress (Himadri et al., 2010; Querido et al., 2012). For example, Himadri et al. (2010) found that high-altitude hypoxic environment leads to activation of *NFκB* in brain of rats. The activated *NFκB* further upregulates the proinflammatory cytokines, such as *IL-1*, *IL-6*, and *TNF-α*. On the other hand, the development of the inflammatory reactions might also be involved in as well as acclimation to high altitudes, while there is a genetic basis to the adaptation of high altitude (Simonson et al., 2010; Yi et al., 2021). In one study, comparing the levels of systemic inflammatory factors in the serum of 36 healthy subjects who had been living on the Qinghai-Tibetan plateau for a short time and 24 subjects who prolonged living on the Qinghai-Tibetan plateau, the authors found that the 36 healthy subjects’ levels of *IL-2*, *IL-3*, *MCP-1*, *IL-1β* in serum were higher than those in the 24 subjects (Yi et al., 2021). Simonson et al. (2010). found that high-altitude adaptation in Tibetans has resulted from local positive selection on the genes of *EGLN1* and *PPARA*. CP is not a local pig breed of Qinghai-Tibetan plateau. In this experiment, the CP were purchased from the plain area of China after weaning. Therefore, the occurrence of intestinal inflammation was observed in the colon of CP-H group, the reason may be that the change of genetic material is also very difficult to achieve in short life of CP in order to adapt to their environment.

Altitude is also an important factor that influence the gut microbiota composition. Most the studies demonstrated that significant differences of gut microbiota were observed among different altitudes (Lan et al., 2017). Researches compared the differences in intestinal microbiota between plateau pika and the low-altitude dauricus. *Scleroderma* was the most abundant genus in the plateau pika, and *Prevotella*, *Oscillospira*, *Ruminococcus*, and *yrc22* were abundant in the plateau pika. While *Proteobacteria*, *Actinobacteria*, and *Verrucomicrobia* were abundant in low-altitude dauricus. The Shannon index of the plateau pika were significantly higher than those of the low-altitude dauricus, and eight genera, including *Streptococcus* and *Pseudomonas*, increased with the altitude (Li et al., 2018, 2019b). Additionally, Zeng et al. (2020). found that *Acinetobacter*, *Pseudomonas*, and *Sphingobacterium* were the top three abundant bacterial genera in high altitude fecal samples in both humans and pigs. In this study, a large variation had also been observed in microbiota composition between CP-H group and CP-L group, including the within community diversity (alpha diversity), the between community diversity (beta diversity) and the microbial composition in the different levels. Therein, the *Terrisporobacter* and *UCG-002* were enriched in the CP-H compared with CP-L. The most likely explanation for this phenotype was that at high altitude, there was a decrease in the barometric pressure and a consequent reduction in the oxygen partial pressure, which lead to the abundances of these obligate anaerobes increased (Moreno-Indias et al., 2015).

Under normal conditions, intact intestinal barrier can inhibit pathogenic bacterial growth (Fukuda et al., 2011). However, some conditional pathogenic bacterial have the opportunity to grow quickly when the barrier is damaged. These pathogenic bacterial destroy the intestinal microecological barrier, then accelerate intestinal damage and result in bacterial antigen translocation from the intestinal cavity to the circulatory system and finally lead to systemic inflammation (Figueredo et al., 2018; Yin et al., 2019). In this study, the abundance of *Terrisporobacter* in CP-H group was significantly higher than CP-L group, and it was positive significant correlation between *Terrisporobacter* and relative mRNA expression levels of *IL-1β*, the LPS content and the activity of DAO. Very little is currently known about *Terrisporobacter*, but there were also indicated that it has been linked to oxidative stress and inflammation in preterm infants (Cai et al., 2019; Ho et al., 2019). The *Terrisporobacter* could produce the urinary toxin, such as trimethylamine-N-oxide (Cai et al., 2019). Trimethylamine-N-oxide may be involved in the pathogenesis of IBD by impacting ATG16L1-induced autophagy and activating NLRP3 inflammasome (Yue et al., 2017; Li et al., 2022). In addition, our data indicate that *Clostridium_ sensu_stricto_1* and *Streptococcus* had higher abundance in colonic chyme of CP-H compared to TP. Meanwhile, *Streptococcus* was significantly positively correlated with D-Lac. In previous studies, *Clostridium_ sensu_stricto_1* and *Streptococcus* are deemed to be opportunistic pathogens associated with colitis (Zou et al., 2020; Wen et al., 2021). Therefore, they may be another important factor to aggravate intestinal inflammation of CP-H in this study. Interestingly, we found that the *Lactobacillus_johnson* and *Lactobacillus_reuteri* were enriched in gut of TP. The *Lactobacillus_johnson* and *Lactobacillus_reuteri* is a well-known gut probiotic (Avall-Jääskeläinen and Palva, 2005; Sung et al., 2018). At present, they have been used as feed additive in swine industry, with very good results (Bampidis et al., 2021). Thus, it may be a good therapeutic candidate for intestinal diseases treatment with the addition of *Lactobacillus_johnson* and *Lactobacillus_reuteri* in the CP-H feed.

Short-chain fatty acids (SCFAs) are produced by anaerobic gut bacteria through saccharolytic fermentation of complex resistant carbohydrates (Muralitharan et al., 2020). The SCFAs predominantly include acetate, propionate, and butyrate which account for approximately 80% of all SCFAs. Therein, butyrate are important substrates for maintaining the colonic epithelium, regulating tight junction proteins and enhancing intestinal barrier function through increasing expression of claudin-1 and ZO-1 (Wang et al., 2012). Studies have reported that the majority of butyrate are produced by a small number of gut bacteria, including *Faecalibacterium prausnitzii*, *Eubacterium rectale*, *Eubacterium hallii* and *Ruminococcus bromi*i (Louis et al., 2010), of which is dominated by *Ruminococcus bromii* (Sun et al., 2017). In this study, no major strains related to butyric acid production were found in the top 15 differential bacterial genera in abundance. Thus, this may be the main reason for the no significant differences found about the concentrations of butyric acid. However, it is worth noting that the concentrations of valerate in CP-H was significantly higher than CP-L and TP. Previous studies found that valerate, which is a SCFAs mainly converted from proteins or amino acids (Gao et al., 2022), was demonstrated that could enhance intestinal barrier functions at physiological concentration (McDonald et al., 2018). However, valerate exhibited the concentration-dependent paradoxical effects on intestinal barrier function. It can exert the opposing effects on normal colonocytes at high concentrations (Burgess, 2012). Interestingly, through the correlation analysis, we found that the concentrations of valerate is significantly positively correlated with *Streptococcus* and *Clostridium_sensu_stricto_1* in this study. Based on our data, we could speculate that the most of the valerate in CP-H group was likely arose from *Streptococcus* and *Clostridium_sensu_stricto_1*. However, the mechanism underlying the production of valerate in gut is not fully understood and further investigation is needed.

## Conclusion

In summary, high-altitude hypoxic environment changed compositions of gut microbiota, promoted the occurrence of colonic inflammation, and disrupted intestinal barrier of the three-way crossbred commercial pigs.

## Data availability statement

The data presented in the study are deposited in the NCBI SRA database with accession number PRJNA 872015.

## Ethics statement

The animal study was reviewed and approved by the Experimental Animal Welfare and Ethical Committee of the Institute of Animal Science, Chinese Academy of Agricultural Sciences.

## Author contributions

CL and GS designed the experiment. CL, GS, JD, and HH carried out the experiment. CL and GS wrote the manuscript. RZ, LC, BW, YZ, HZ and ZW revised the manuscript. All authors contributed to the article and approved the submitted version.

## Funding

This research was supported by the Tibet Science and Technology Projects (XZ-2019-NK-NS-003) and Agricultural Science and Technology Innovation Program (CAAS-ZDRW202006-02, ASTIPIAS07).

## Conflict of interest

The authors declare that the research was conducted in the absence of any commercial or financial relationships that could be construed as a potential conflict of interest.

## Publisher’s note

All claims expressed in this article are solely those of the authors and do not necessarily represent those of their affiliated organizations, or those of the publisher, the editors and the reviewers. Any product that may be evaluated in this article, or claim that may be made by its manufacturer, is not guaranteed or endorsed by the publisher.
